# Systematic review of interventions to increase recruitment and retention of black, minority and ethnic patients into randomised controlled trials

**DOI:** 10.1186/1745-6215-14-S1-P83

**Published:** 2013-11-29

**Authors:** Gulnaz Iqbal, Janet Dunn, Margaret Thorogood

**Affiliations:** 1 University of Warwick, Coventry, UK; 2 University of the Witwatersrand, Johannesburg, South Africa

## 

Recruitment into cancer trials has been an area of concern for numerous years. In 2000 the NHS plan sought to double the number of patients entering cancer trials within 3 years. This was achieved through the creation of the National Cancer Research Network in 2001. By 2004, approximately 10.9% of all cancer patients were entered into a trial. However, recruitment of Black, Minority and Ethnic (BME) patients into clinical trials has been reported to be under-represented compared to the split of ethnicity both within the reported incidence of disease and the reported total population. Mason found a two-fold under representation of South Asians within breast cancer trials (Mason *et al*, 2003).

In the USA, a significant under-representation of Hispanics, Blacks and Asians was found when comparing ethnic proportions recruited within oncology trials with existing cancer cases (Murthy *et al*, 2004). The under-representation of any patient group within a clinical trial, specifically an ethnic one, can bias trial results, and subsequent extrapolation into the general population.

A systematic literature review of interventions to improve the recruitment and retention of minority patients into clinical trials was conducted. The search intended to capture literature pre- and post the Race Relations Act Amendment (2000) and the USA National Institute of Health Revitalisation Act (1993).

Preliminary results of the review have revealed a paucity of published evidence from the UK, with the majority of the articles meeting the inclusion criteria originating from the USA. Final results will be presented.

